# DNA Damage, Cell Death, and Alteration of Cell Proliferation Insights Caused by Copper Oxide Nanoparticles Using a Plant-Based Model

**DOI:** 10.3390/biology13100805

**Published:** 2024-10-09

**Authors:** Sazada Siddiqui

**Affiliations:** Department of Biology, College of Science, King Khalid University, Abha 61413, Saudi Arabia; sasdeky@kku.edu.sa

**Keywords:** nanoparticles, CuO, cytotoxicity, genotoxic, *Pisum sativum* L.

## Abstract

**Simple Summary:**

Nanoparticles (NPs) have received significant consideration because of their special properties and valuable applications in numerous segments. In the current era, the fast growth of NP manufacturing and their plentiful uses puts the environment at additional risks. To explicate the noxious effects of NPs, numerous plant bioassays have been proposed. Since *Pisum sativum* L. has complex mitotic dynamics, it is a test plant used to assess the genotoxic effects of environmental pollutants. Using the *Pisum sativum* test system, this work was designed to assess the toxicity potential of CuO NPs by morphological (SG and RL) and genotoxic (CD, MI, CKP, MNF, and CAF) methods. Even though cytogenetic analyses of environmental pollutants in plants have been reported previously, this is the first work of its type to demonstrate the morphological and genotoxic effects of CuO NPs in *Pisum sativum*. These studies are very essential, since the pea is a vital food grain crop and a tremendous source of ayurvedic medications and manure, as well as its use as a vegetable.

**Abstract:**

The speedy growth of copper oxide nanoparticle (CuO NP) manufacturing due to their wide application in industries has caused concerns due to their increased discharge into the environment from both purposeful and accidental sources. Their presence at an elevated concentration in the environment can cause potential hazards to the plant kingdom, specifically to staple food crops. However, limited research is available to determine the consequences of CuO NPs. The present study aimed to assess the morphological and cytological changes induced by CuO NPs on *Pisum sativum* L., a key staple food crop. Seeds of *Pisum sativum* were exposed to various concentrations of CuO NPs (0, 25, 50, 75, 100, and 125 ppm) for 2 h, and their effects on seed germination (SG), radicle length (RL), cell proliferation kinetics (CPK), mitotic index (MI), cell death (CD), micronucleus frequency (MNF), and chromosomal aberration frequency (CAF) were studied. The results indicate a significant reduction in SG, RL, CPK, and MI and a significant dose-dependent increase in CD, MNF, and CAF. CuO NP treatment has led to abnormal meiotic cell division, increased incidence of micronucleus frequency, and chromosomal aberration frequency. Additionally, the CuO NP-treated groups showed an increase in the percentage of aberrant meiotic cells such as laggard (LG), double bridge (DB), stickiness (STC), clumped nuclei (CNi), precocious separation (PS), single bridge (SB), and secondary association (SA). CuO NP treatment led to reductions in SG as follows: 55% at 24 h, 60.10% at 48 h, and 65% at 72 h; reductions in RL as follows: 0.55 ± 0.021 cm at 24 h, 0.67 ± 0.01 cm at 48 h, and 0.99 ± 0.02 cm at 72 h; reductions in CPK as follows: 34.98% at prophase, 7.90% at metaphase, 3.5% at anaphase, and 0.97% at telophase. It also led to a 57.45% increase in CD, a 39.87% reduction in MI, and a 60.77% increase in MNF at a higher concentration of 125 ppm. The findings of this study clearly show that CuO NPs have a genotoxic effect on the food crop plant *Pisum sativum.*

## 1. Introduction

Nanoparticles (NPs) have received significant consideration because of their special properties and valuable applications in numerous segments [1,2,3]. In the current era, the fast growth of NP manufacturing and their plentiful uses puts the environment at additional risks. NPs are categorized as organic or inorganic. Inorganic NPs are classified into metal oxides and metals. To raise the figures of inorganic NPs with novel applications, uses, and characteristics, the amount of funding and research work has been raised enormously. Generally, metal oxide NPs like iron and copper (oxides), zinc, and titanium are the most used NPs in consumer products. The most vital metal oxide NPs are CuO NPs, which are applied in agronomic industries, ecological remediation, the food, chemical, medical, and textile industries, plastics, paint coatings, cosmetics, fuel additives, electronics, and the treatment of wastewater [4,5]. CuO NPs’ widespread application in electronics, energy, optics, antimicrobial agents, gas sensors, catalysis, and cross-coupling reactions has sparked a commercial revolution [6]. Safety and ecological concerns are mostly significant for CuO NPs due to their extensive usage in fungicides, wood preservatives, and aquatic antifouling paints, and their toxicity and higher redox activity have been stated in in vitro studies [7]. The buildup of NPs in groundwater and soil might possibly lead to their buildup in plant tissues. CuO NPs are highly noxious to plants and humans [8]. NPs might translocate from the roots to aboveground tissues after entering plants through the roots. CuO NPs were not present in the center cylinder of rice, which means that the Casparian strip serves as the last apoplastic barrier separating the vascular tissue from the outer plant [9,10]. The stomatal openings may allow for the air-dispersed NPs to penetrate and move through [11,12]. CuO NPs influenced the germination rate, transpiration rate, photosynthesis, reduced shoot and root length, damaged roots, biomass, and quality of yield in the following species: *Brassica chinensis*, *Zea mays*, and *Triticum aestivum* [13,14,15]. CuO NPs affect biochemical plant contents, as indicated by a reduction in chlorophyll content and lipid peroxidation and enhanced hydrogen peroxide causing a rise in reactive oxygen species (ROS) generation in cucumbers [16]. The impact of CuO NPs extends to the DNA level by triggering DNA damage in grasses, *Raphanus sativus*, and *Cucumis sativus* [17,18,19].

To explicate the noxious effects of NPs, numerous plant bioassays have been proposed. Since *Pisum sativum* L. has complex mitotic dynamics, it is a test plant used to assess the genotoxic effects of environmental pollutants [20,21,22,23]. Using the *Pisum sativum* test system, this work was designed to assess the toxicity potential of CuO NPs by morphological (SG and RL) and genotoxic (CD, MI, CKP, MNF, and CAF) methods. Even though cytogenetic analyses of environmental pollutants in plants have been reported previously, it is the first work of its type to demonstrate the morphological and genotoxic effects of CuO NPs in *Pisum sativum*. These studies are very essential, since the pea is a vital food grain crop and a tremendous source of ayurvedic medications and manure, and it is used as a vegetable [24]. It is a commonly used leguminous plant and is an essential source of dietetic protein.

## 2. Materials and Methods

### 2.1. Nanoparticle (NP) Characterization

CuO NPs were characterized using a scan electronic microscope functioning at a steady accelerating voltage of 10 kV. After ultrasonically sonicating NPs in ethanol, a drop from it was applied to stub with a layer of carbon-conducting, double-sided sticky tape (SPI Supplies^®^, West Chester, PA, USA). Using an X-ray diffractometer, with a scintillation counter probe SC-30, the particle size was further confirmed. CuO NPs were immediately put on sample holders, and using Cu k α radiation (=0.15406 nm), the X-ray diffraction (XRD) array was documented at room temp. in a span of 2 θ = 20°–70° with a step of 0.02° and 2° min^−1^ speed. Applying Scherrer’s formula and considering the first three peaks of the acquired pattern, particle size was calculated, and figures were analyzed by Traces software 3.0 (AAC-Aerodynamic Aerosol Classifier, Cambustion Ltd., Cambridge, UK).

### 2.2. Procurement of Seeds and Chemical

The research was conducted at the Dept. of Biology, King Khalid University in Abha, Saudi Arabia. The National Seed Corporation (NSC) in New Delhi, India provided the pea seeds. CuO NPs having purity of 99.5%, particle size < 50 nm, and surface area 45 ± 10 m^2^/g were procured from Sigma-Aldrich, Saint Louis, MO, USA.

### 2.3. Preparation of CuO NP Solution

CuO NPs were suspended in distilled water and dispersed for 30 min using ultrasonic vibration (100 W, 40 kHz). To avoid particle aggregation, before usage, the suspension was stirred by applying tiny magnetic bars.

### 2.4. Treatment of Seeds with CuO NPs

To assure surface sterility, healthy and fresh seeds were treated with 5% NaOCl for 10 min for surface sterilizing and thoroughly rinsed with distilled water multiple times. Seeds were subsequently treated for 2 h with six concentrations (0, 25, 50, 75, 100, and 125 ppm) of CuO NP suspension, and 50 seeds in each Petri plate, having a size of 200 × 20 mm in triplicate, were left on wet cotton to germinate at a regulated temperature of 25 ± 1 °C for the next 72 h in the dark. Distilled water was used as a negative control. Newly emerged roots 1–2 cm in length were used in the experiment (mitotic index, cell proliferation kinetics, and cell death). The complete experiment was carried out three times under the same conditions.

Another batch of 75 seeds in three replicates (25 seeds per replicate) per dose was sown in earthen pots (48 cm length and 28 cm width) in a greenhouse for studying chromosomal anomaly frequency (CAF) and micronucleus frequency (MNF) in pollen mother cells (PMCs). For comparison, non-treated control plants were also cultivated. 

### 2.5. Determining Seed Germination and Radicle Length

Thirty seeds that were presoaked in water were kept with proper spacing in each of the Petri plates, which were concealed with two layers of filter paper and then soaked in 10 mL of CuO NP solution having varied doses (25 to 125 ppm). For comparison, a control without exposure was grown. Experiments were carried out in the growth chamber and kept in the dark for 72 h for seed germination. Following this, a 16/8 h light/dark photoperiod was created having a light intensity of 500 L mol/m^2^/s. A humidity of 60% and temperature of 25 ± 1 °C were maintained throughout the experiment. Radicle formation was used to determine seed germination. Every 24 h, the length of newly generated roots was measured with a millimeter ruler. The whole experiment was conducted three times under identical settings. The formula for calculating seed germination is as follows:

$$\mathrm{Seed}\;\mathrm{germination}\;\%=\frac{\mathrm{Number}\;\mathrm{of}\;\mathrm{germinated}\;\mathrm{seeds}}{\mathrm{Total}\;\mathrm{number}\;\mathrm{of}\;\mathrm{seeds}\;}\times 100$$

### 2.6. Cytotoxicity Evaluation in Root Tip Cells of Pisum Sativum 

#### Fixing of Roots and Analysis of Cell Proliferation Kinetics (CPK) and Mitotic Index (MI)

A cytotoxicity experiment was carried out on root tips of seeds exposed to 25 to 125 ppm with CuO NPs. For comparison, a control group without treatment was used. Roots were gathered and soaked for 24 h in fixation solution (ethanol–glacial acetic acid, 3:1) before being shifted to 70% ethanol and kept in a refrigerator until microscopic examination. For preparing each slide, 2 roots from each sample were hydrolyzed for 10 min in 1 N HCl before being dyed for 10 min in 2% acetic orcein. MI preparations from root tips were carried out as stated by Qian et al. [25] with slight changes [23]. A total of 1000 cells from every sample were examined to determine MI, which is a ratio of dividing cells. CPK frequencies were calculated by dividing the number of cells in every division stage by the total number of mitotic cells. All mitotic cells were scrutinized by a light microscope (LM) having an oil immersion (100×). Each slide was scrutinized and coded. The following formula is used for calculating MI.

$$\mathrm{Mitotic}\;\mathrm{index}\;\%=\frac{\mathrm{Number}\;\mathrm{of}\;\mathrm{cells}\;\mathrm{in}\;\mathrm{mitosis}\;}{\mathrm{Total}\;\mathrm{number}\;\mathrm{of}\;\mathrm{cells}\;}\times 100$$

### 2.7. Detection of Cell Death (CD) in Root Tips (RTs)

Trypan blue dye (non-permeable) uptake was used to evaluate cell death in RTs. Only the dead cell membrane can be permeated by trypan blue. RTs (0.1 cm) were submerged in trypan blue (0.4%, *w*/*v*) at room temperature for 15 min, then rinsed twice with 2.5 g/mL chloral hydrate solution for 10 min each, using the method stated by [26]. After the samples were prepared, an optical microscope (Olympus CX23, Olympus Co., Ltd., Tokyo, Japan) was used to analyze and capture images. 

### 2.8. Bud Collection and Fixation

Through control, flower buds were collected, and plants were grown from seeds treated with CuO NPs at 34 days following SG. Flower buds were fixed in Carnoy’s soln. for 40 min. before being shifted to propionic acid that was saturated with ferric acetate for 24 h and stored in 70% alcohol. A treatment of 0.5% propionocarmine was used to squash the anthers. Permanent slides were made using the normal butanol alcohol (NBA) series, mounted in Canada balsam, and dried at 45 °C.

### 2.9. Genotoxicity Evaluation

#### 2.9.1. Chromosomal Aberration Frequency (CAF) Analysis in Pollen Mother Cells (PMCs)

Chromosomal aberrations in metaphase and anaphase plates were examined by LM. A total of 50 metaphase and anaphase plates from each slide were studied for laggard (LG), single bridge (SB), double bridge (DB), clumped nuclei (CNi), stickiness (STC), precocious separation (PS), and secondary association (SA). The formula for calculating chromosomal aberration frequency is as follows:

$$\mathrm{Chromosomal}\;\mathrm{abnormality}\;\%=\frac{\mathrm{Number}\;\mathrm{of}\;\mathrm{abnormal}\;\mathrm{cells}\;}{\mathrm{Total}\;\mathrm{number}\;\mathrm{of}\;\mathrm{cells}\;\mathrm{observed}}\times 100$$

#### 2.9.2. Micronucleus Frequency (MNF) Analysis in Pollen Mother Cells (PMCs)

For MNF assessment, 100 cells from each slide were scored to compute MNF. Micro-nucleated cells were examined using a binocular light microscope (Olympus, Tokyo, Japan) (100×). MNF was scored using the technique stated by Tolbert et al. [27]. 

### 2.10. Data Analysis

One-way ANOVA using SPSS software (version 16.0, SPSS Inc., Chicago, IL, USA) was applied to find the significance of differences in variables. The changes were statistically significant at *p* < 0.05. All the outcomes are expressed as mean ± standard error.

## 3. Results

### 3.1. Nanoparticle (NP) Size Determination

**Figure 1:**
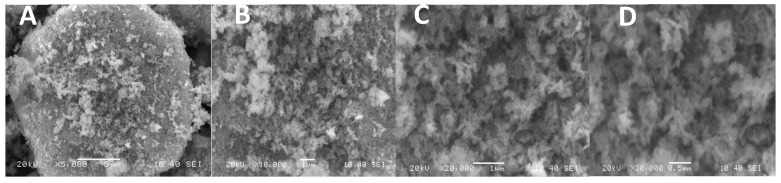
SEM image of CuO NPs at a magnification of ×5000 (**A**), ×10,000 (**B**), ×20,000 (**C**) and ×30,000 (**D**).

**Figure 2:**
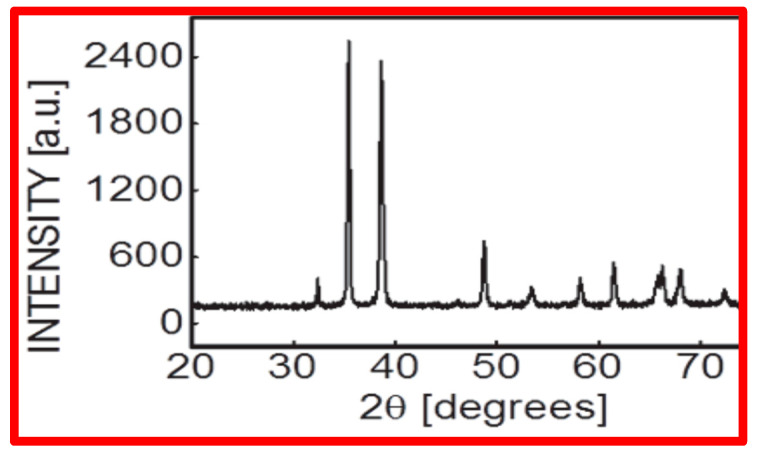
X-ray diffraction pattern of CuO NPs recorded at room temperature in the range 2θ = 20°–70° with a step of 0.02°, speed = 2°min^−1^ using Cu kα radiation (ʎ = 0.15406 nm).

CuO NPs were found to be spherical in shape and <50 nm in size, according to NP characterization investigations applying SEM (scanning electron microscopy) [Figure 1A–D], which used Scherer’s method to analyze XRD (X-ray diffractometry) data (Figure 2).

### 3.2. Effect of CuO NPs on Seed Germination (SG) of Pea

**Figure 3:**
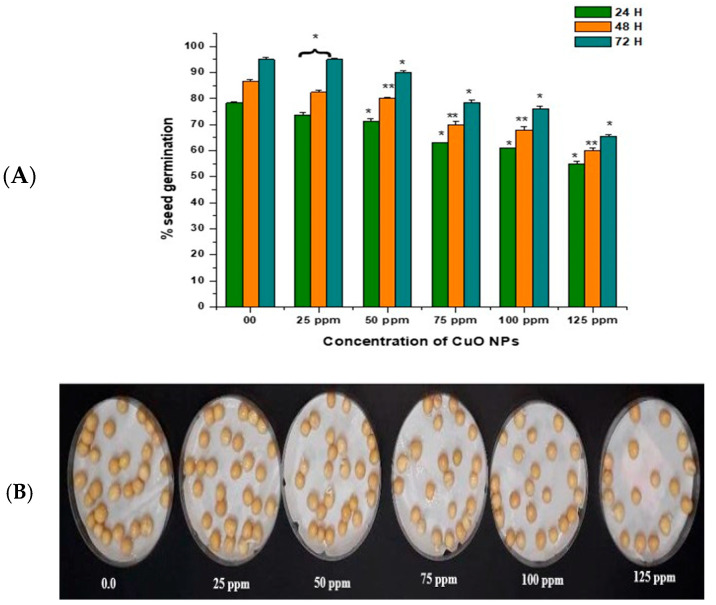
(**A**,**B**). Effect on SG of pea treated with CuO NPs for 2 h. * *p* < 0.05; ** *p* < 0.01. % are mean of three replicates ± SE, 0.0 = control group.

In the control group, seeds treated with double-distilled water (DDW) water for 2 h produced 78.40% of SG at 24 h, which rose to 86.55% and 95.11% at 48 and 72 h (Figure 3A,B). CuO NP exposure from 25 ppm to 125 ppm for 2 h resulted in a significant reduction in SG (*p* < 0.05) at 24 h, with reductions of 73.76% at 25 ppm, 71.43% at 50 ppm, 63.00% at 75 ppm, 61.00% at 100 ppm, and 55.00% at 125 ppm. CuO NP exposure from 25 ppm to 125 ppm for 2 h resulted in a very significant decrease in SG (*p* < 0.01) at 48 h, with decreases of 82.43% at 25 ppm, 80.20% at 50 ppm, 70.00% at 75 ppm, 68.00% at 100 ppm, and 60.00% at 125 ppm. CuO NP exposure from 25 ppm to 125 ppm for 2 h resulted in a significant decrease in SG (*p* < 0.05) at 72 h in comparison to control, with decreases of 89.10% at 25 ppm, 90.00% at 50 ppm, 78.53% at 75 ppm, 76.15% at 100 ppm, and 65.6% at 125 ppm.

The maximum SG was reported in seeds treated with 25 ppm CuO NPs (about 73.76% at 24 h; 82.43% at 48 h; and 89.10% at 72 h after treatment). The lowest SG was reported in seeds treated with 125 ppm CuO NPs (about 55.00% at 24 h; 60.10% at 48 h; and 65.6% at 72 h after treatment). Overall, a significant reduction (*p* < 0.05; *p* < 0.01) in SG was reported in all time intervals in a dose-dependent manner.

### 3.3. Effect of CuO NPs on Radicle Length (RL) of Pea

**Figure 4:**
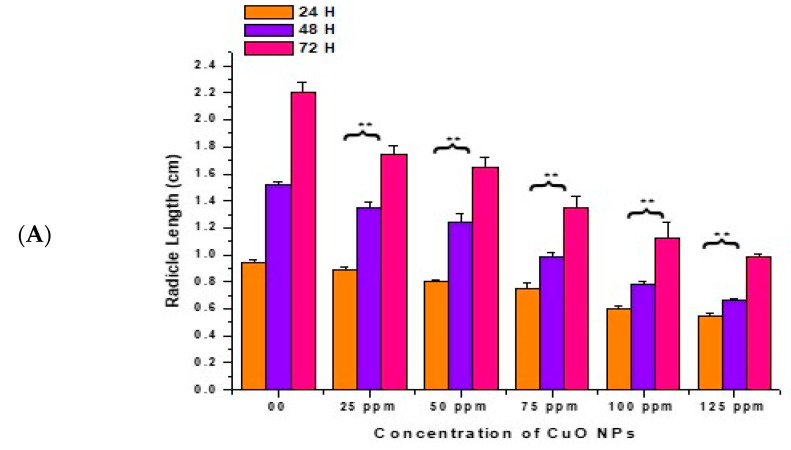

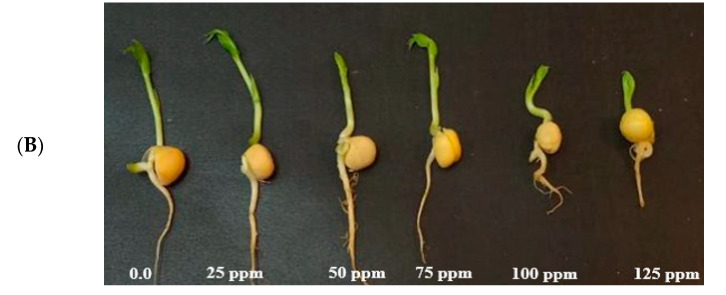
(**A**,**B**). Effect on RL of pea treated with CuO NPs for 2 h. ** *p* < 0.01. Data are mean of three replicates ± SE, 0.0 = control group.

In the control, the RL increased with a surge in time (0.94 ± 0.020 cm) at 24 h, (1.52 ± 0.021 cm) at 48 h, and (2.21 ± 0.07 cm) at 72 h (Figure 4A,B). At 24 h of CuO NP exposure for 2 h, a very significant (*p* < 0.01) decrease in RL was reported in seeds exposed to 25 ppm (0.89 ± 0.023 cm), 50 ppm (0.80 ± 0.020 cm), 75 ppm (0.75 ± 0.040 cm), 100 ppm (0.60 ± 0.025 cm), and 125 ppm (0.55 ± 0.021 cm), respectively, in comparison to the control.

At 48 h of CuO NP exposure for 2 h, at 25 to 125 ppm, a very significant (*p* < 0.01) decrease in RL was noticed, with decreases of (1.35 ± 0.04 cm) at 25 ppm, (1.24 ± 0.07 cm) at 50 ppm, (0.99 ± 0.03 cm) at 75 ppm, (0.78 ± 0.02 cm) at 100 ppm and (0.67 ± 0.01 cm) at 125 ppm. At 72 h of CuO NP exposure for 2 h, at 25 to 125 ppm, a very significant (*p* < 0.01) decrease in RL was noticed, with decreases of (1.75 ± 0.06 cm) at 25 ppm, (1.65 ± 0.07 cm) at 50 ppm, (1.35 ± 0.09 cm) at 75 ppm, (1.12 ± 0.12 cm) at 100 ppm, and (0.99 ± 0.02 cm) at 125 ppm in comparison to the control. The lowest RL was recorded at 125 ppm at 24 h (0.55 ± 0.021 cm), at 48 h (0.67 ± 0.01 cm), at 72 h (0.99 ± 0.02 cm). The maximum RL was noticed at 25 ppm (0.89 ± 0.023 cm) at 24 h, (1.35 ± 0.04 cm) at 48 h, and (1.75 ± 0.06 cm) at 72 h.

### 3.4. Effect of CuO NPs on Cell Proliferation Kinetics (CPK) of Pea

**Figure 5:**
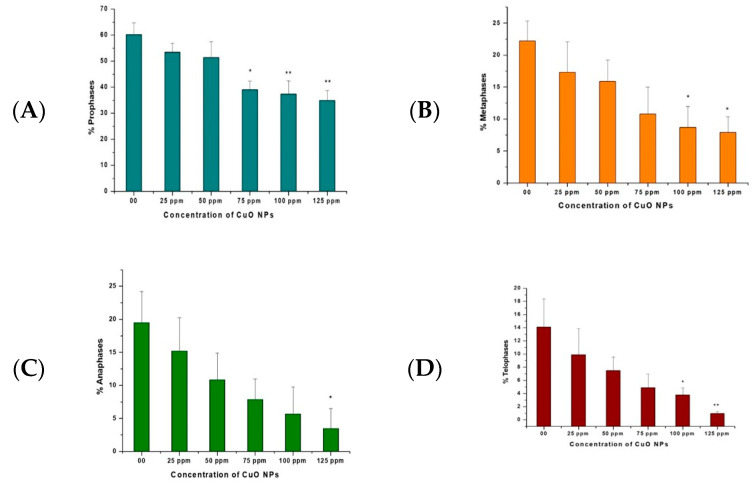
(**A**–**D**). Effect on CPK of pea treated with CuO NPs for 2 h. * *p* < 0.05; ** *p* < 0.01. Data are mean of three replicates ± SE, 0.0 = control group.

The effects of CuO NPs on CPK of pea root tip cells (RTCs) are depicted in Figure 5A–D. In the control, treatment with DDW for 2 h showed a rise in prophase, metaphase, anaphase, and telophase. A marginal decline was reported at 25 ppm to 125 ppm of CuO NP-treated root tips for 2 h.

A significant decrease (*p* < 0.05) was reported in prophase at 75 ppm (39.12%); in metaphase at 100 ppm (8.70%) and at 125 ppm (7.90%); in anaphase at 125 ppm (3.50%); and in telophase at 100 ppm (3.80%). A very significant decrease (*p* < 0.01) was detected in prophase at 100 ppm (37.34%) and 125 ppm (34.98%) and in telophase at 125 ppm (0.97%).

### 3.5. Effect of CuO NPs on Mitotic Index (MI) of Pea

**Figure 6:**
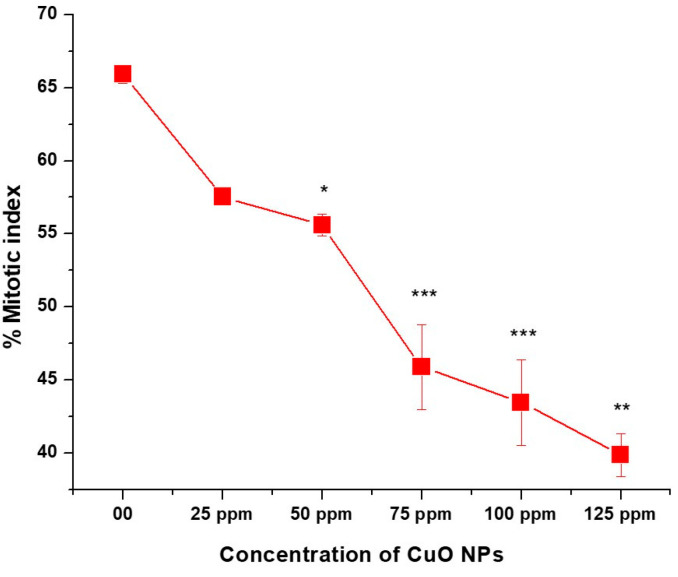
Effect on MI of pea treated with CuO NPs for 2 h. * *p* < 0.05; ** *p* < 0.01; *** *p* < 0.001. Data are mean of three replicates ± SE, 0.0 = control group.

The effect of CuO NPs on MI of *P*. *sativum* RTCs is depicted in (Figure 6). In the control group, the MI was 65.90% in DDW-treated seeds for 2 h. A marginal decline (non-significant) in MI was detected in CuO NP-treated seeds for 2 h at 25 ppm, with a decrease of (57.53 ± 53), and a significant decrease (*p* < 0.05) was detected at 50 ppm, with a decrease of (55.60 ± 0.745). At 75, 100 and 125 ppm, a very significant inhibitory effect (*p* < 0.01) on MI was reported when treated for 2 h with CuO NPs, with values of approximately (45.88 ± 2.92; 43.45 ± 2.92 and 39.87 ± 39.87). A significant (*p* < 0.001) decrease in MI was noticed in seeds treated with 25 ppm to 125 ppm of CuO NPs for two hours as compared to the control group. The lowest MI was measured at 125 ppm (39.87 ± 0.025), and the highest MI was recorded at 25 ppm (57.53 ± 0.41). Overall, significant decreases (*p* < 0.05; *p* < 0.01) in MI were reported in a dose-dependent manner.

### 3.6. Effect of CuO NPs on Cell Death (CD) in Root Tip Cells (RTCs) of Pea

**Figure 7:**
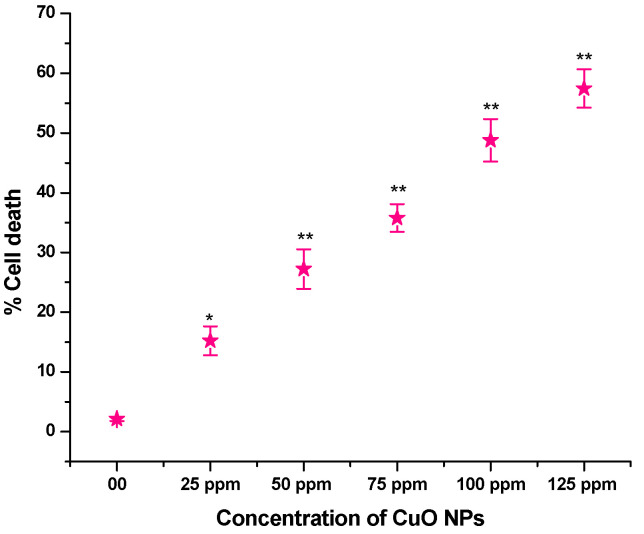
Effect on CD of pea treated with CuO NPs for 2 h. * *p* < 0.05; ** *p* < 0.01. % are mean of three replicates ± SE, 0.0 = control group.

The effect of CuO NPs on CD in pea RTCs is depicted in Figure 7. In the control, the CD was 2.12% in seeds exposed to DDW for 2 h. A significant increase (*p* < 0.05) in CD was detected in seeds exposed to CuO NPs for 2 h at 25 ppm, with an increase of 15.23%. At 50 to 125 ppm, a very significant increase (*p* < 0.01) in CD was reported when treated for 2 h with CuO NPs, with increases of 27.23% at 50 ppm, 35.77% at 75 ppm, 48.77% at 100 ppm and 57.45% at 125 ppm in comparison to the control. The lowest CD was measured at 25 ppm (15.23%), and the highest CD was recorded at 125 ppm (57.45%). There is a dose-dependent increase in CD. 

### 3.7. Effect of CuO NPs on Micronucleus Frequency (MNF) of Pea in Pollen Mother Cells (PMCs)

**Figure 8:**
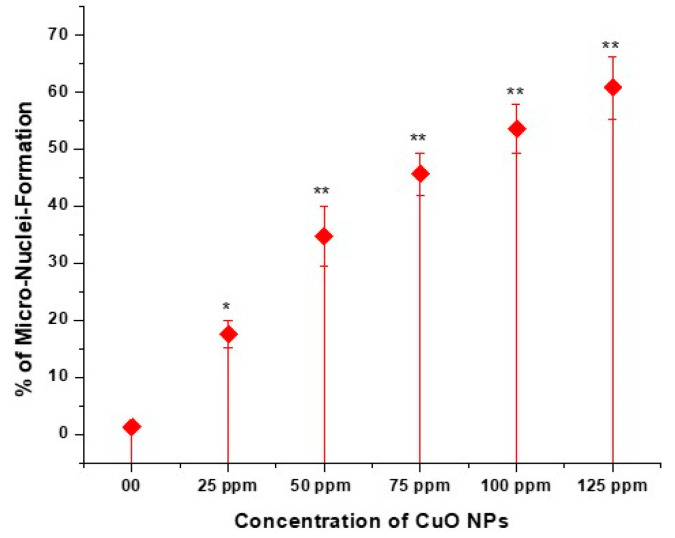
Effect on MNF of pea treated with CuO NPs for 2 h. * *p* < 0.05; ** *p* < 0.01. % are mean of three replicates ± SE, 0.0 = control group.

**Figure 9:**
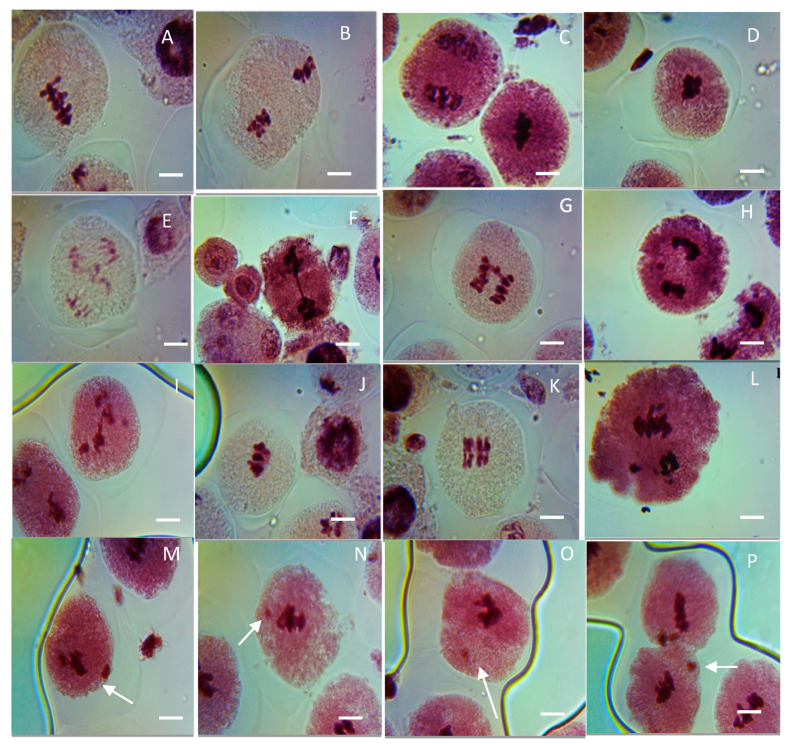
Representative images of various meiotic aberrations observed in PMCs of CuO NP-exposed pea. (**A**,**B**) Clumped nuclei (CNi) at metaphases I and II; (**C**,**D**) stickiness (STC) at metaphases I and II; (**E**–**G**) bridges (BR) at anaphases I and II; (**H**,**I**) laggards (LG) at anaphases I and II; (**J**,**K**) secondary association (SA) at metaphase I and metaphase II; (**L**) precocious separation (PS) at anaphase II; micronucleus (**M**–**P**). Scale bars = 10 μm.

The effect of CuO NPs on the MNF of peas in PMCs is depicted in Figure 8 and Figure 9 (M-P). In the control, the MNF was 1.25% in PMCs exposed to DDW for 2 h. A significant increase (*p* < 0.05) in MNF was detected in PMCs exposed to CuO NPs for 2 h at 25 ppm, with an increase of 17.56%, and a very significant increase (*p* < 0.01) in MNF was observed when treated for 2 h with CuO NPs, with increases of 34.77% at 50 ppm, 45.67% at 75 ppm, 53.55% at 100 ppm, and 60.77% at 125 ppm, in comparison to the control. The lowest MNF was measured at 25 ppm (17.56%), and the highest MNF was recorded at 125 ppm (60.77%). The increase in MNF was dose-dependent. 

### 3.8. Effect of CuO NPs on Chromosomal Aberration Frequency (CAF) of Pea in Pollen Mother Cells (PMCs)

In the control, no aberrant metaphase–anaphase plates were reported except FG (0.01%) in PMCs of pea (Table 1, Figure 9A–L). Different types of chromosomal aberrations, such as LG (laggard, H,I), DB (double bridge, E), STC (stickiness, C,D), CNi (clumped nuclei, A,B), PS (precocious separation, L), SB (single bridge, F,G), and SA (secondary association, J,K), were reported in metaphase–anaphase plates. In seeds treated with CuO NPs for 2 h, there is an increase in the ratio of aberrant metaphase–anaphase plates with a rise in CuO NP doses. Cytological studies reveal that the level of CAF steadily increased with rising concentrations of CuO NPs for 2 h. Studies of diverse phases of meiotic division show that every phase of division was affected. The percentage of formation of LG, DB, PS, SB, and SA were reported to be the maximum at 125 ppm: LG (1.24%, significant *p* < 0.05), DB (0.98%, significant *p* < 0.05), PS (0.91%, significant *p* < 0.05), SB (0.86%, significant *p* < 0.05), SA (1.10%, very significant *p* ≤ 0.01) at 125 ppm, and STC (1.66%, significant *p* < 0.05), CNi (0.98%, significant *p* < 0.05) at 100 ppm, as compared to the control group. In addition, the minimum percentage of chromosomal abnormalities were reported at 25 ppm SB (0.15%, significant *p* < 0.05), SA (0.25%, not significant) at 2 h of CuO NP-treated seeds. The increased incidence of CAF was in the following order for 2 h CuO NP treatment: STC > LG > SA > DB > CNi > PS > SB.

## 4. Discussion

Concerns related to the consequences of nanoproducts on human health, agricultural plants, and the environment have grown in the preceding few decades due to their increasing use. According to Zhang et al. [28], the seed germination test has developed into a direct, quick, and accurate biological test technique for determining phytotoxicity. Due to their solubility and possibility of discharging metal ions, nanoparticles can have harmful or more complex effects on the environment.

Numerous studies have documented diverse impacts of different Cu-based NPs on plants. Hafeez et al. [29] suggest that Cu NPs may improve wheat growth and yield. Yang et al. [30], Duran et al. [31], and Wang et al. [32] found no evidence of inhibition of seed germination of maize, common bean, or *Arabidopsis thaliana* by Cu-based NPs. However, the germination of tomatoes [33], cucumbers [34], *Lactuca,* and *Raphanus* [35] was markedly suppressed by exposure to CuO NPs. Additionally, *Brassica juncea* [36], eggplant [37], and *Brassica rapa* [38] showed reduced plant growth when exposed to CuO NPs. Lower doses of CuO NPs may be advantageous for growing rice seedlings, as indicated by Tiwari et al. [39]; however, larger concentrations of CuO NPs severely decreased the growth of seedlings. 

The toxicity of CuO NPs is associated with disruptions to plant metabolism, decreased levels of sugar, total chlorophyll, and elevated levels of lipid peroxidation. These factors lead to a notable decrease in the inhibition of root growth and plant biomass in *A. thaliana* [40] and *A. rapa* [38]. According to Hossain et al. [41], CuO NPs caused damage to root surface cells, increased the buildup of ROS-disturbed defensive systems, and ultimately led to reduced growth in rice. 

Green pea seedlings exposed to the highest concentration of CuO NPs (500 mg dm^−3^) showed a considerable reduction in total chlorophyll content, and this might have been caused by the excess Cu present because of CuO NP treatment [42]. The histological and anatomical parameters of pea seeds exposed to 10–100 mg/L declined with an evident reduction in elasticity of the epidermis and mesophyll, and hence, the outcomes revealed CuO NP toxicity regarding pea seeds at doses higher than 10 mg/L and the inhibitory effects on pea seed germination at a dose of 100 mg/L [43]. Pea plants were grown in soil modified with either bulk CuO, CuCl_2_ or nanoCuO plus IAA at 10 and 100 μM, and factorial analysis revealed that IAA decreased the number of plants. Furthermore, IAA in combination with ionic Cu decreased the number of stems, leaves, root lengths, carotenoids and the production of chlorophyll [44]. 

Similar results were noticed in the current investigation when seeds were exposed to CuO NPs at concentrations ranging from 25 ppm to 125 ppm. This resulted in delays in *P. sativum* seed germination during intervals of 24 h, 48 h, and 72 h. It might be one of the factors responsible for inhibiting seed germination in the present study.

According to the findings of our study, seedlings grown when treated with CuO NPs showed altered development as compared to seedlings grown from untreated plants. The germination and development of seedlings at lower NP concentrations may be because of increased uptake and imbibition of oxygen and water, which may have accelerated the action of protease and amylase enzymes, amino acids, and soluble sugars that are needed for the germination and development of seedlings. Higher concentrations, on the other hand, could have caused particle agglomeration, which could have hampered oxygen and water uptake and hence influenced germination [45].

Zhang et al. [28] stated that after treating spinach seeds with TiO_2_ NPs, there is a rise in water absorption, thereby augmenting the rate of germination. Uptake of water is a crucial mechanism in seed germination. NPs at appropriate doses may form new pores in the seed coat, facilitating water uptake by the embryo, which is required to start growth and cellular metabolisms [46]. A report found that when compared to controls, lower doses enhance root growth. This could be due to the seedlings’ increased uptake of nutrients and water at lower concentrations [47]. The decrease in survival of seedlings could be attributed to physiological abnormalities and impediments in the various metabolic actions of cells [15].

An adequate quantity of copper, one of the essential nutritional components, can stimulate plant development. Sharma et al. [48] stated that excessive copper negatively affects plants. According to Ogunkunle et al. [49], NPs may be able to pass through the endodermis and cortex layer and gather in the cells that make up the central cylinder of roots. This might be described by the fact that CuO NPs could be directly absorbed by roots and then transported to shoots [50,51]. According to Liman et al. [52], the buildup of CuO NPs in the roots and shoots of cowpea caused it to become phytotoxic to those parts of the plant in a concentration-dependent way. Numerous plants have been shown to exhibit increased lignification of the roots with excessive nanoparticle treatment, which results in roots growing more slowly [40]. The current study’s findings showed that RL increases with varying time intervals at lower concentrations of 25 ppm to 75 ppm, while RL declines with varying time intervals at higher concentrations of 100 ppm to 125 ppm, at 24 h, 48 h, and 72 h, respectively. Therefore, the growth inhibition of CuO NPs at higher doses may be due to excess Cu buildup by *Pisum sativum*. Furthermore, hormonal abnormalities and DNA damage may be the cause of the suppression of root growth under CuO NP stress [53,54]. According to numerous earlier studies, plants’ osmotic equilibrium is altered because of heavy metal stress [46]. 

According to the results of the proportions of distribution of precise mitotic stages, CuO NPs decreased the percentage of prophase, metaphase, anaphase and telophase at all concentrations dose-dependently. The findings are consistent with those of [55,56]. The findings imply that a decrease in the percentage of prophase, metaphase, anaphase, and telophase at all doses, and hence MI, could be because of the seizure of one or more mitotic stages or a reduction in the pace of cell development [57,58].

CuO NPs showed cytotoxicity in this study by reducing the MI dose-dependently. This demonstrates that CuO NPs have a mito-depressive effect in the pea. Various studies have shown that a decrease in cell action might be because of the variations in the duration of the mitotic cycle. Some authors have ascribed the mitotic inhibition to a rise in the duration of the S phase [59,60]. A reduced percentage of MI may result from disrupted cell cycle progression, such as G1-S and S-M phases, with exposure to CuO NPs. According to Das et al. [61], NPs can access DNA through nuclear pores or when the nuclear membrane dissolves in cells going through mitotic division. According to Jing-Jing [59], the MI reacts to the multiplying capability of a region where cells divide actively, but it diminishes during exposure to hazardous substances showing cytotoxicity.

Based on our findings, there is a dose-dependent decrease in CD in root tips of peas treated with CuO NPs at all concentrations ranging from 25 to 125 ppm. Plants utilize vacuolar content and vacuoles in two ways for programmed cell death (PCD): destructively and non-destructively [44]. The collapse of the vacuolar membrane leads to the discharge of vacuolar hydrolytic enzymes in cytosol, which in turn causes direct and instant cell death. It is unknown whether CuO NP exposure interferes with root cell energy generation and thus impacts the growth of roots. Mitochondria, as energy-producing organelles, assist cells in performing their division, function, and elongation via the metabolism of energy [62]. Hence, it is considered that the observed reduction in root growth in this study is due to the CuO NP-induced death of root cells, particularly due to deficient energy generation in root cells [63]. CuO NPs reduced the micronutrient contents in *Brassica napus* seedlings and instigated cell death and callose production [64].

CuO NP-induced genotoxicity was further demonstrated by the existence of micronuclei in the PMC cells of pea. The number of MN is drastically raised with a rise in concentrations from 25 to 125 ppm following a 2 h treatment with CuO NPs. It is still unclear how exactly NPs cause micronuclei to form. On the other hand, acentric fragments or lagging chromosomes that are not included in the complete genetic complement during mitosis result in the development of micronuclei. According to Hosseinpour et al. [65], the nuclear membrane mimics the nucleus proper as it organizes around the excluded region. MN generation suggested a mutagenic effect ensuing from unrepaired damage or incorrectly repaired damage in parental cells [66], caused by CuO NPs. Other researchers have documented analogous genotoxic effects of CuO NPs and *Vicia faba*, *Glycine max*, *Allium cepa*, and *Triticum sativum* [67,68].

Analysis of genotoxicity can be achieved effectively through the induction of chromosomal changes. In this study, alterations in chromosomal behavior caused by NPs have been examined to evaluate the range of chromosomal damage. CAs were absent in the control plant, but even at the lowest concentration, treated plants displayed a variety of anomalies, including LG, SB, DB, STC, PS, CNi and SA. The incidence of these anomalies indicates their aneugenic, tubergenic and clastogenic effects [69,70], which are caused by disorders in the spindle apparatus (anomalous metaphase, telophase, anaphase and bridges) and breaking of chromosomes (fragments, micronuclei) [71].

One possible explanation for the occurrence of these anomalies in the root tips is that the NPs enter the plant through RTCs, interfering with usual cell division and causing noxiousness. A dependable constraint for determining the frequency of the cell division is MI. In this study, cdk genes that restrict cdc2 expression were the cause of the reduction in MI with rising doses. This is a normal stress reaction that results in extended S-phase progression and cell cycle arrest [72]. NPs have the potential to produce genotoxicity by direct interactions with nucleic acids, disruption of protein assembly during DNA replication, or formation of ROS, as noted by Ochoa et al. [73]. Prior research on both plant and animal cells has also shown that NPs build up in the cytoplasm and enter sub-cellular structures like the nucleus and mitochondria, where they cause DNA breaks because it boosts ROS generation by the disruption of the mitochondrial respiratory chain.

## 5. Conclusions

The cytotoxicity of NPs was directly proportional to the dose of exposure. Higher doses of CuO NPs exhibit a detrimental effect on the genetics and morphology of plants, implying that environmental NPs are easily absorbed by plants and eventually make their way up the food chain. CuO NPs are a potential threat to the environment, as by inducing oxidative stress, they inhibit plant growth, consequently triggering DNA damage. Regarding the disposal of NPs and the doses at which they become hazardous, these findings are noteworthy. These results call for additional research on the mechanism of NP transport in plants, the effects of ecotoxicology on biochemical constraints, and their probable repercussions in the food chain.

## Figures and Tables

**Table 1:** Effect of CuO NPs on CAF of pea after 2 h exposure.

CuO NP Concentration	LG	DB	STC	CNi	PS	SB	SA	TA
00.00	0.00 ± 0.00	0.00 ± 0.00	0.00 ± 0.00	0.00 ± 0.00	0.00 ± 0.00	0.00 ± 0.00	0.01 ± 0.001	0.01 ± 0.001
25 ppm	0.00 ± 0.00	0.00 ± 0.00	0.00 ± 0.00	0.00 ± 0.00	0.00 ± 0.00	0.15 ± 0.003 *	0.25 ± 0.03	0.40 ± 0.033
50 ppm	0.00 ± 0.00	0.38 ± 0.01 *	0.34 ± 0.01 *	0.00 ± 0.00	0.00 ± 0.00	0.26 ± 0.09 *	0.35 ± 0.01	1.33 ± 0.12
75 ppm	0.65 ± 0.06 *	0.55 ± 0.05 *	0.96 ± 0.05 *	0.25 ± 0.03 *	0.35 ± 0.03 *	0.46 ± 0.03 *	0.75 ± 0.03	3.97 ± 0.28
100 ppm	0.97 ± 0.05 *	0.76 ± 0.02 *	1.66 ± 0.63 *	0.98 ± 0.10 *	0.68 ± 0.05 *	0.66 ± 0.05 *	0.98 ± 0.21 *	6.68 ± 1.11
125 ppm	1.24 ± 0.20 *	0.98 ± 0.10 *	1.50 ± 0.99 *	0.97 ± 0.23 *	0.91 ± 0.23 *	0.86 ± 0.11	10 ± 0.42 **	7.56 ± 2.28

* *p* < 0.05; ** *p* < 0.01 compared to control group. % mean of three replicates ± SE, 0.0 = control group. LG, laggard; DB, double bridge; STC, stickiness; CNi, clumped nuclei; PS, precocious separation; SB, single bridge; SA, secondary association; TA, Total aberration.

## Data Availability

The data that support the findings of this study are available from the corresponding author upon reasonable request.
